# Folklore use of medicinal plants for the treatment of gynecological diseases in Pakistan-a review

**DOI:** 10.1016/j.heliyon.2024.e34869

**Published:** 2024-07-19

**Authors:** Salihah Khadim, Khafsa Malik, Abeer Kazmi, Tahira Sultana, Amir Ali, Khalid Mehmood, Rizwan Ul Hassan, Muhammad Nasir Bashir, M. Mahmood Ali

**Affiliations:** a Department of Botany, PMAS, Arid Agriculture University, Rawalpindi, Pakistan; b The State Key Laboratory of Freshwater Ecology and Biotechnology, The Key Laboratory of Aquatic Biodiversity and Conservation of Chinese Academy of Sciences, Institute of Hydrobiology, Chinese Academy of Sciences, Wuhan, 430072, China; c University of Chinese Academy of Sciences, Beijing, 100049, China; d Department of Biology, PMAS, Arid Agriculture University, Rawalpindi, Pakistan; e Department of Chemical & Biological Engineering, Gachon University (13120) 1342 Seongnamdaero, Sujeong-gu, Seongnam-si, Gyeonggi-do, Republic of Korea; f Department of Mechanical Engineering, Yonsei University, Seoul, 120-749, Republic of Korea; g Department of Mechatronic Engineering, Atlantic Technological University Sligo, Ash Lane, F91 YW50 Sligo, Ireland

**Keywords:** Ethnopharmacology, Gynecological diseases medicinal plants, Part used

## Abstract

**Background:**

Gynecological issues and sexually transmitted infections (STIs) pose significant challenges to women's health, particularly in developing nations. These challenges are exacerbated by limited access to modern reproductive healthcare facilities, economic constraints, and entrenched cultural norms. Consequently, most of the Pakistani population relies on traditional ethno-medicinal healthcare systems. This preference stems from the ease of access, affordability, widespread availability, and inherent trust placed in these alternative healthcare methods.

**Aim/objective:**

The inquiry aimed to report details on the application of conventional uses of plants in the health field in rural areas that could contribute to advancing the natural discovery of drugs. The objective of this analysis is to provide researchers with information on conventional and empirical knowledge of plant species concerning women's diseases.

**Methodology:**

Information on the common use of medicinal plants in treating women's diseases was gathered from electronic databases. As a keyword for the quest, ethnobotany, and ethnopharmacology were used together with gynecological complications.

**Result:**

The work of the current analysis has revealed that 217 plant species belonging to 89 families have been used in Pakistan's rural communities. The majority of plant species belong to the Apiaceae family, followed by the Asteraceae, Fabaceae, Solanaceae, Rosaceae, Lamiaceae, and other families. The biological interpretation of plants used in rural communities of Pakistan revealed that herbs and trees are the dominant forms with 58 % and 23 % respectively while shrubs and sub-shrubs with a low percentage of 17 % and 2 %. In natural preparation, leaves 29 %, flowers 22 %, seeds 14 %, fruits 14 %, roots 13 %, bark 7 %, and stems 5 % were the most used parts respectively and aerial parts, dried pericarp, bulb, bud, berry, latex, wood, rhizome, husk, fruit coat, oil, resins, twigs, and shoot were also used in minimum percentage. A multitude of plant species have found extensive application in the management of diverse women's health issues. These encompass concerns such as fatigue, mood fluctuations attributed to menstrual problems, gonorrhea, complications related to pregnancy, cravings for specific foods, throbbing breast pain, abdominal and pelvic cramps, excessive vaginal discharge, mastitis, irritability, abortion-related matters, headaches, uterine hemorrhage, Menorrhagia, Amenorrhea, Menopause, Vomiting Abortion, infertility and lactation challenges, as well as the regulation of lochia flow.

**Conclusion:**

This review provides remarkable information about the use of medicinal plants against women's diseases in the rural communities of Pakistan. It opens the gateway for the discovery of natural drug development.

## 1 Introduction

In conventional healing systems, man has relied on wild medicinal plant resources [1,2]. Different application modes and uses have been adopted by indigenous people to leverage these natural resoures [[3], [4], [5]]. Since then, many farming people around the world have turned to wild plants for food and medicine [[6], [7], [8]]. In many developing countries, local populations still rely on plant-based medicines today, while the global healthcare system is primarily dependent on plant-based ingredients [[9], [10], [11], [12]]. The use of plants in the supply of folk medicines for the health care system, as well as a means of food for the needy and rural populations, is unavoidable [10,13].

Research into the ethnomedicinal uses of plants by indigenous people is also notable, offering a key to the study of the new source of herbal drugs [[14], [15], [16]]. The use of medicinal plants to cure various illnesses is as ancient as human civilization [4,17,18]. Plant programs for humanity are not limited to food, clothes, and shelter, but they are also well known for their use in health care, decoration, and spiritual ceremonies [18,19]. Approximately 20 % of all plants present in this world are used to cure diseases in humans for medicinal purposes [13,20].

Herbal therapy has a long practice tradition. The value of these medicinal herbals has also been illustrated by our society and civilization. Medicinal plants now are of the same importance as they were centuries ago [5,[21], [22], [23]]. Even today, in rural areas, herbal medicine plays an important role and numerous locally manufactured medicines are still being used as household remedies for various ailments [24]. Medicinal plants provide rural areas with primary health services and nearly 80 % of rural people are projected to be dependent on them. Herbal medicine appears to have a stronger focus on promoting health and preventing illnesses rather than solely concentrating on curing them [25]. Depletion of medicinal plants happens due to the destruction of natural habitat by overgrazing and exploitation [26]. In allopathic medicines, extracts of medicinal plants are also used. The young generation, though, is now entirely oblivious to the use and benefits of these species [27,28].

Ethnobotany deals with the study of plant contact and human culture; it provides the views of customs and scientific knowledge of local people [11,29]. Ethno-gynecology is a common technique used by indigenous tribes to discuss problems regarding women's physical condition. Therapies used to cure gynecological issues such as miscarriage, menstrual pain, menopause, morning sickness, leucorrhea, delivery complications, and others, are treated through the use of medicinal plants [30,31].

Cultural traditions such as herbal steam baths, dietary limitations, and body signals [26,[32], [33], [34], [35], [36], [37], [38], [39], [40], [41], [42]] are popular in many Asian countries during the postpartum era, and several medicinal plants are used in these practices [42].

Pakistan has a rich flora with 1572 genera and 5521 species in total, most of which are limited to the regions of Hindukush, Himalaya, and Karakorum [43]. Women in Pakistan's rural communities are well-versed in the conventional use of medicinal plants. Rural women choose conventional plant medicine for their illnesses due to a shortage of modern facilities [[44], [45], [46], [47], [48]]. As one of the big non-timber forest products (NTFPs) [49], people gather about 600 medicinal plant species. Twenty-eight [31] leading herbal processing units use medicinal plants to produce numerous preparations, including 75 extensively manufactured crude herbal products. Around 60,000 traditional practitioners (Hakeems, practitioners of traditional medicine) use medicinal plants as household remedies for treating many diseases in rural and remote areas [50]. Local populations have decades of indigenous knowledge and experience that have been passed from generation to generation concerning plants in their regions [6]. Around 84 % of the world's population relied on traditional drugs in the early 1950s; however, this trend is now restricted to rural areas [51,52].

The high cost of traditional allopathic medicine in Pakistan, as well as the inaccessibility of modern healthcare facilities, particularly in remote areas, contributes to the country's dependence on herbal medicine. Furthermore, particularly in rural areas, traditional medicine is often believed to be a safer care choice [53,54]. In rural and urban areas around the country, there are 60,000 herbalists (Hakims) and 10,000 homeopathic doctors who use rudimentary medicinal plants in their clinics. Ten percent of Pakistani plants are used as medicinal items. Moreover, based on their knowledge and ancestral recommendation, rural area dwellers use the plants [55,56]. Approximately 80 % of Pakistan's population lives in rural areas where these plants are readily visible [57]. Herbal recipes are still the pillar of health care in the rural communities of this country, in the form of traditional customs and rituals [58,59].

Gynecological problems are frequently faced by rural women in Pakistan due to hunger, poverty, unhygienic living conditions, and hard physical labor. In every village, women locally called 'Daiya' are found and specialize in phytotherapy to relieve gynecological problems with indigenous medicinal plants [60]. The maintenance of ethno-gynecological information is also a necessity [61]. Today, allopathic medications, anti-inflammatory drugs, non-steroidal analgesics, and anesthesia are used more commonly to treat gynecological issues. These medications are successful but also include surgical/anesthetic-related side effects such as vomiting and nausea; sexual issues following hysterectomy; stomach problems or skin rashes; and, more importantly, drug-related kidney, liver, and heart failure, especially when drugs are used for a long time. In addition, certain medications can damage the embryo when used in pregnancy [62]. Ethnogynecology is a recently emerging ethnobotany branch that discusses the use of medicinal plants to treat gynecological problems (e.g., abortion, menstrual problems, leucorrhoea, antifertility, and delivery problems) [[63], [64], [65], [66]].

There is also little research on the use of ethnomedicine in the treatment of gynecological conditions by rural women. Furthermore, due to the rise of allopathy and recent modernization, consciousness is declining, as the millennial generation has no interest in learning about these beneficial practices and curing techniques.

This review aims to consolidate existing literature regarding Pakistani medicinal plants employed in the treatment of gynecological issues. Additionally, it delves into local nomenclature, specific plant parts utilized, preparation methods, and traditional applications, all of which contribute to the identification of promising medicinal plant candidates for subsequent in-vitro, in-vivo, and phytochemical studies.

## 2 Materials and methods

### 2.1 Study area

**Fig. 1:**
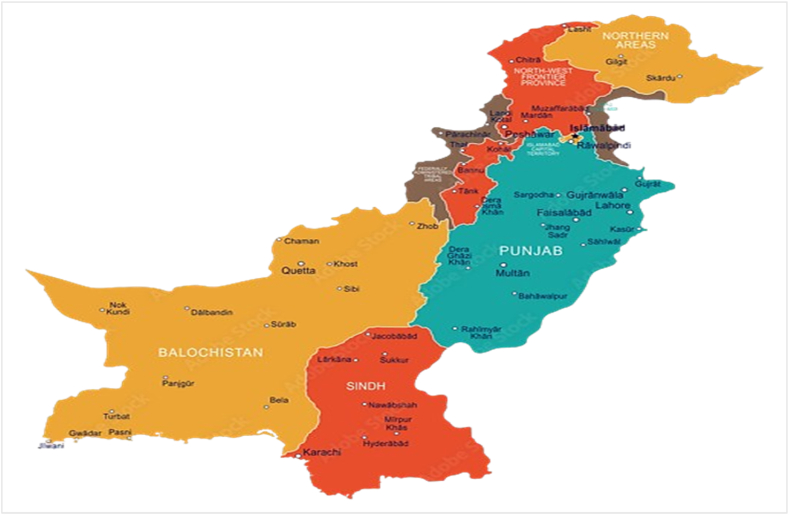
Map of selected study area (Pakistan).

Pakistan has a total area of 803,950 km and is situated on a strategic route between 24° and 37° north latitude and 62° and 75° east longitude [67,68] (Fig. 1). India is situated in Pakistan's east, while Iran is located in the west. China and Afghanistan are found in Pakistan's north and northwest [69], respectively. The Arabian Sea, with a 146 km long coastal corridor, is located in the south of Pakistan. Its climatic spectrum spans from arid regions in the west to subtropical zones in the east, which has contributed to a wide array of soil types conducive to agricultural activities. The nation's cultural tapestry is a rich mosaic shaped by its historical legacy and the pluralism of religious traditions, with Urdu serving as its official language. Pakistan grapples with significant challenges in the realms of education and socioeconomic disparities, particularly in rural areas, although concerted efforts have been made to enhance access to quality education and healthcare services. The country's history is steeped in the legacies of ancient civilizations, British colonialism, and the complexities of post-independence political dynamics.

### 2.2 Methods for data collection

The data was gathered from previously published data in journals, websites, databases, and the Pakistani traditional uses of medicinal plants. Science Direct, PubMed, Wiley, Scopus, Google Scholar, National Thesis Center, and Springer databases were searched for the necessary publications on gynecological problems and potent herbs. As a keyword for quest, ethnobotany, and ethno-pharmacology were used together with gynecological complications.

## 3 Results and discussion

### 3.1 Composition and growth forms of medicinal plants used in herbal

The results of the latest study confirm the folklore usage of medicinal plants in rural Pakistani cultures to treat women's diseases. According to the current study, 217 species from 89 families have been used in Pakistani rural populations (Table 1). The study revealed a diverse array of plant species utilized for various purposes in rural communities of Pakistan. Among the families, Apiaceae stood out as the most prominent, with a total of 19 plant species identified for their traditional applications. This was closely followed by the Asteraceae family with 17 species, highlighting its significance in local herbal practices. Other notable plant families included Fabaceae with 12 species, Lamiaceae with 11, and Solanaceae with 8 species. Several families, such as Rosaceae, Amaranthaceae, Brassicaceae, Moraceae, Malvaceae, Nyctaginaceae, Poaceae, Verbenaceae, Anacardiaceae, and Euphorbiaceae, were also represented with multiple plant species. The wide diversity of plant families and species underscores the rich traditional knowledge of medicinal plants in these rural communities and calls for further research to explore the therapeutic potential of these plants through scientific validation and clinical studies. According to previous literature, these families were the most often used in conventional medicine against women's diseases in Pakistani rural societies.

**Table 1:** Major bioactive compounds in dominant families.

Sr. No	Family Name	Major Bioactive Compounds
1	Apiaceae	α-pinene, β-pinene, β-phellandrene, carvacrol, limonene, menthol, menthone, 1,8-cineole, *cis*-ocimene, niloticin, (l)-fenchone, thymol and eugenol
2	Asteraceae	Quercetin, luteolin fisetin, 3-hydroxyflavone, kaempferol, (−)-epicatechin, (−)-epigallocatechin and bractein
3	Fabaceae	Apigenin, daidsein, genistein, carotenoids, lectins, tricin saponins, terpenoids, butrin, derrubone, and castanospermine
4	Lamiaceae	Thymol, 1,8-cineole, eugenol, α-cadinol, α-terpineol, pulegone, Linalool, and somenthone
5	Solanaceae	Solanine, soalasonine, tomatine, chaconine, solamargine, incanumine, isocapsicastrine, khasianine and solaradixine
6	Amaranthaceae	Phenol, sterol, terpenoid, coumarin, tannin, chlorogenic acid, polyphenols, and quercetein

### 3.2 Growth form of plants used in Pakistan's traditional medicine

**Fig. 2:**
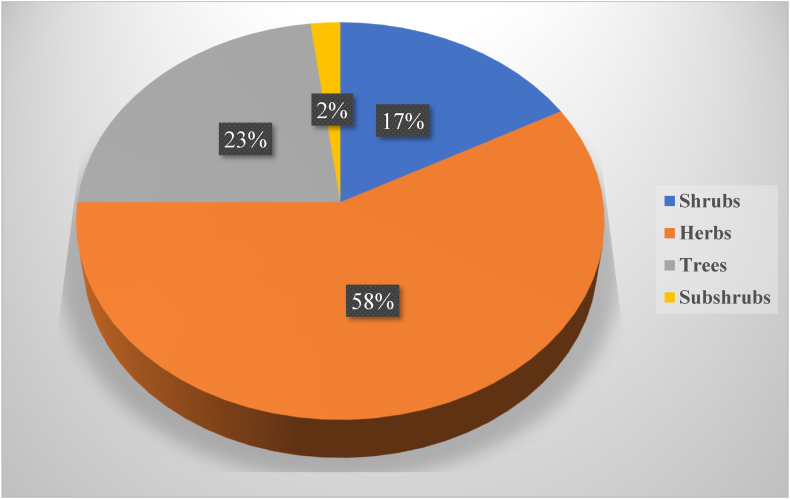
Percentage contribution by each plant growth form of the study area to traditional medicinal uses.

Herbs and trees are the most common types of plants in Pakistani rural areas, accounting for 58 percent and 23 percent of all plants used (Fig. 2). There were also shrubs and subshrubs with low percentages of 17 percent and 2 %, respectively, mentioned (Table 2).

**Table 2:** List of traditionally used medicinal plants and their parts for the treatment of women's diseases.

Family	Botanical name (genera/species)	Voucher No.	Vernacular name	Part use	Ethnomedicinal uses	Ref.
Malvaceae	*Abelmoschus esculentus* (L.) Moench	200,013,666	Bhindi	Fruits	Gonorrhea	[70]
	*Abutilon indicum* (L.) Sweet	200,013,676	Koso beta	Whole plant	Gonorrhea, Leucorrhoea, gynae, Abortion	[70]
	*Sida cordifolia* L.	200,013,759	Drojakay	Seeds	Sexual weakness	[3,58,63,70]
	*Malva parviflora* L.	242,416,823	Tikalai	Leaves	Menses	[70]
	*Hibiscus rosa-sinensis* L.	200,013,716	Shoe flower	Flower	Cure white discharge and treat irregular periods	[71]
Fabaceae	*Acacia farnesiana* (L.) Wight & Arn.	242,353,819	Vilayati kikar tree	Gum	Leucorrhoea	[70]
	*Acacia modesta* Wall.	250,063,319	Palusa & Phulai	Whole plant	Backache after delivery, Gonorrhea, gynae, Increased sexuality, body and muscle pain, Tonic after delivery	[49,58,63,66,[70], [72], [73]]
	*Acacia nilotica* (L.) Delile	200,011,856	Kikar tree	Leaves, Bark, Pod	Gynae, gonorrhea, Leucorrhea, Female impotency	[70]
	*Bauhinia variegata* L.	200,011,962	Kachnar	Flowers & Buds	Enhance lactation	[63]
	*Butea monosperma* (Lam.) Taub.	242,309,402	Palay	Gums, Seed, Root & Bark	Backache, weakness, gonorrhea and leucorrhoea	[3,63,66,[73], [74], [75], [76], [77]]
	*Caesalpinia bonduc* (L.) Roxb.	200,011,969	Katranj & nata	Seeds	Puerperal after delivery, leucorrhea, menstrual disorders	[58,66]
	*Cassia fistula* L.	200,012,019	Granjawan	Fruit	Easy delivery	[42]
	*Cytisus scoparius* (L.) Link	242,316,730	Genestra	Whole plant	Easy delivery	[58]
	*Erythrina variegata* L.	N/A	Flame trees	Leaves	Cure irregular periods	[71]
	*Lotus corniculatus* L.	200,012,202	Rub, Fathi Khani	Whole plant	Pregnancy, Backache, urinary tract infection, and genital tonic	[66,70]
	*Medicago laciniata* var. brachycantha	242,331,812	Karari	Seeds	Internal wounds	[42]
	*Medicago sativa* L.	200,012,215	Malkindye	Leaves & Stem	Menses	[70]
	*Glycyrrhiza glabra* L.	242,323,657	Khawazha walee	Stem	During delivery, gastric lavage encourages the expulsion of "dead blood" and lochia	[42]
	*Prosopis cineraria* (L.) Druce	242,341,383	Jandi or Jand or kunda	Leaves, Bark, Flowers, & Pods	Heal cuts, use for birth control, blood or protein loss, and painful menstruation	[78]
Amaranthaceae	*Aerva lanata* (L.) Juss. ex Schult.	242,426,440	Shutpask	Roots	Abnormal stoppage of menses	[58]
	*Achyranthes aspera* L.	200,006,961	Kurshaka, Putkanda, & Geshkay	Whole plant	Gonorrhea and painful delivery	[3,49,58,[75], [76], [77],]]
	*Amaranthus caudatus* L.	200,006,980	Ganiar	Leaves	Leucorrhoea	[58]
	*Amaranthus spinosus* L.	200,006,989	Azghkay	Whole plant	Amenorrhea	[66]
	*Amaranthus viridis* L.	200,006,991	Ghanarr, Gunhar, & Chalverai	Whole plant	Control menstruation and leucorrhoea	[63,66,73,79]
	*Dysphania ambrosioides* (L.) Mosyakin & Clemants	242,414,750	Benakkai	Leaves	Relieve postpartum discomfort and increase milk supply in breastfeeding mothers.	[63,70]
Asteraceae.	*Achillea millefolium* L.	200,023,010	Yarkand & Boodko	Leaves, Aerial parts	Leucorrhea, gestational diabetes, anemia, menstrual pain, menopausal hot flashes	[61,66]
	*Artemisia parviflora* Waldst. & Kit.	200,023,298	Kharkalich	Seed	Abdominal pain, menstrual disorders	[66]
	*Artemisia sieberi* Besser	200,023,298	Deranna	Aerial parts	Abdominal pain	[61]
	*Artemisia vulgaris* L.	200,023,371	Berenjasf & Chaagu	Flower & leaf	Amplification of uterine spasm, carminative, sedative, helpful to suppress menses	[61,71]
	*Calendula arvensis* L.	250,005,003	Damberguly	Flower	Menstrual pain and prolonged menstrual periods	[66]
	*Calendula officinalis* L.	220,002,139	Hamishe bahar	Flower	Irregular menstrual	[61]
	*Conyza canadensis* (L.) Cronquist	200,023,708	Paleet	Whole plant	Painful menstruation	[58]
	*Echinops echinatus* Roxb. ex Royle	N/A	Kanderi bhattar	Roots	Sexual insufficiency	[58]
	*Eclipta alba* (L.) Hassk.	242,319,736	Sofed banghra & Skha botay	Whole plant	Miscarriage	[58,63,70]
	*Hertia intermedia* (Boiss.) Kuntze	N/A	Goongapat & Manguli	Leaves	Galactagogue is a natural remedy for reducing breast swelling and discomfort, and menstrual cycle issues	[42,64]
	*Lactuca serriola* L.	242,328,083	Kahu	Leaves	Increase milk flow	[58,63,70]
	*Matricaria chamomilla* L.	242,331,663	Baboneh	Flower	Menstruation additive,	[61]
	*Pulicaria undulata* (L.) C.A.Mey.	N/A	Bomadarane balochi	Flower & Leaf	Nausea and menstruation additive	[61]
	*Vernonia cinerea* (L.) Less.	200,024,619	Seh devi	Seeds	used in amenorrhea, gonorrhea, and female sterility	[79]
	*Tagetes erecta* L.	200,024,576	Satveerga, Gainda, & Nacha gulay	Leave, & Roots	Muscular pain & swelling of the body, and irregular menstruation	[58,63,70,80]
	*Microcephala lamellata* (Benth.) Benth.	220,008,538	Chargualai	Flower & Whole plant	Strengthen the uterus, swelling of the uterus	[42]
Ranunculaceae	*Aconitum heterophyllum* Wall. ex Royle	242,420,118	Patris, Bhang dewana, & Sarba wali	Latex & Root	Gynecology	[70]
	*Nigella sativa* L.	242,426,411	Kalwangi	Seeds	Lactiferous and aphrodisiac	[66]
Araceae	*Acorus calamus* L.	200,027,130	Skhwaja	Rhizome	Irregular menstruation	[63,70,77]
	*Cocos nucifera* L.	200,027,077	Cofra	Fruit	Lactiferous	[66]
	*Phoenix dactylifera* L.	200,027,092	Kajor & Khormag	Fruit	Anemia, backache, aphrodisiac sexual tonic, to strengthen the backbone, and waist pain	[42,66]
Pteridaceae	*Adiantum capillus-veneris* L.	200,003,518	Alphy butty & Kohay botay	Whole plant	Used in gynecological problems	[63,75,81]
	*Adiantum venustum* D.Don ex Wall.	200,003,555	Pata, Kakwa, & Paroshan	Leave	Healing of wound, abnormal stoppage of menses	[58,80]
Lamiaceae	*Ajuga parviflora* Benth.	242,420,254	Bugle	Leaves & Roots	Amenorrhea	[66]
	*Ajuga bracteosa* Wall. ex Benth.	200,019,466	Bhutti	Whole plant	Amenorrhea	[63]
	*Lallemantia royleana* (Benth.) Benth.	200,019,747	Malangyan	Whole plant	Gastric, constipation, and postpartum pain	[42]
	*Mentha viridis* L.	230,003,673	Podina	Leaves	Menses	[70]
	*Mentha spicata* L.	200,019,821	Pahari podina	Leaves	Easy delivery	[58,70,&59]]
	*Mentha longifolia* (L.) L.	200,019,816	Velanay	Leaves	Easy food Digestion	[63]
	*Nepeta cataria* L.	200,019,873	Mutrich	Whole plant	Delayed menstruation	[58]
	*Ocimum basilicum* L.	200,019,914	Neazboo/naezboi	Seeds	After arrival, encouraging the expulsion of "dead blood" and lochia	[42]
	*Thymus serpyllum* L.	N/A	Mervezei	Whole plant	Menses and gynae	[70]
	*Origanum vulgare* L.	200,019,922	Ban ajwain	Leaves	Menstrual pain	[58]
	*Ziziphora tenuior* L.	200,020,502	Gharpodina	Whole plant	Gastric	[42]
Alliaceae & Amaryllidaceae Alliaceae	*Allium cepa* L.	200,027,457	Piaz, Pimaz	Bulb, Seed	Slim the abdomen, Aphrodisiac, and menstrual pain	[13,42,49,61,70]
	*Allium sativum* L.	200,027,526	Tom, Uzha	Bulb	After delivery, blood cleansing (to promote the expulsion of "dead blood" and lochia) constipation after giving birth	[42]
Amaryllidaceae	*Allium schoenoprasum* L.	200,027,528	Pemlok	Leaf, Seed	Aphrodisiac, infection	[61]
Asphodelaceae	*Aloe vera* (L.) Burm.f.	200,027,555	Zuqam	Leaves	Contraceptive, abortifacient, launch menses, and amenorrhea	[66]
Apiaceae	*Ammi visnaga* (L.) Lam.	200,015,348	Spairkay	Fruit	Regulate the menses and uterus Infection	[66]
	*Anethum graveolens* L.	200,015,349	Shot'k	Leaf fruit	Lactiferous, carminative menstruation additive, and menopause hot flashes	[61]
	*Bunium persicum* (Boiss.) B.Fedtsch.	250,063,037	Torazira	Seeds	During birth, galactagogues, postpartum hurts, and lochia facilitates the expulsion of "dead blood."	[42]
	*Bupleurum falcatum* L.	250,063,037	Ziwarbotay	Whole plant	Help in menses regulation	[66]
	*Carum carvi* L.	200,015,475	Zire	Fruit	Aphrodisiac, lactiferous diuretic, and irregular menstrual	[61]
	*Carum copticum* (L.) Benth. & Hook.f. ex Hiern	242,421,175	Ezbootk & Ajghowan	Fruit	Menstruation additive, carminative, lactiferous, diuretic, gestational, and hypertension	[61]
	*Coriandrum sativum* L.	200,015,503	Geshnizh	Fruit	Diuretic and gestational edema	[61]
	*Cuminum cyminum* L.	200,015,512	Spin zira	Seeds	Postpartum pain, gastric	[42]
	*Dorema ammoniacum* D.Don ex Loud.	220,004,338	Hoshtarak & Ooshi	Gum	Abortion and aphrodisiac	[42,61]
	*Dorema aureum* (Spach) Thell. ex Britton	250,063,131	Oshtork	Gum	Abortion and aphrodisiac	[61]
	*Daucus carota* L.	200,015,518	Ghasoon, Gajar, & Gazara	Seed	smooth delivery, abnormal stoppage of menses	[28,31,58,63,70]
	*Foeniculum vulgare* Mill.	200,015,555	Khawazha walani, Kagilani, Raz, & Kagu	Seeds, Leaves, & Fruit	Postpartum pain, galactagogue, Menses pain, vomiting, regulate the menses, lactiferous, Menstruation additive, and menopause hot flashes,	[42,61,63,66,73,75]
	*Ducrosia anethifolia* (DC.) Boiss.	N/A	Goatk	Aerial parts & seed	Carminative, menstruation additive, lactiferous	[61]
	*Ferula assa-foetida* L.	250,063,132	Peterk	Leaf, gum, resin	Abortion and infection	[61]
	*Eryngium biehersteinianum* Pomel	N/A	Yakandaz	Whole plant	Backache and erotic tonic	[66]
	*Pimpinella anisum* L.	200,015,767	Raz	Fruit	Lactiferous, carminative menstruation additive, and menopause hot flashes	[61]
	*Psammogeton biternatum* (Bunge) Pimenov & Kljuykov	220,011,032	Sparkai	Seeds	Postpartum infections	[42]
	*Heracleum candicans* Wall. ex DC.	200,015,565	Skhwara	Roots	Help in menses regulation	[66]
	*Trachyspermum ammi* (L.) Sprague	242,426,007	Sparakai, Ajwain & Sperkay	Seeds	After birth, promoting the expulsion of "dead blood" and lochia, irregular postpartum discomfort, and gastric reflux	[42,58,70,71,82]
Acanthaceae	*Justicia adhatoda* L.	220,006,995	Baikar	Root, Leaves	Gynae, abortifacient, and leucorrhoea	[63,70]
Aizoacaeae	*Trianthema portulacastrum* L.	220,013,693	Itsit	Whole plant	Abortion	[58]
Anacardiaceae	*Schinus molle* L.	220,012,109	Toor maruch tree	Bark, leaf, & fruits	Regulate menses	[70]
	*Mangifera indica* L.	200,012,696	Anbe	Fruit	Aphrodisiac	[61]
	*Pistacia terebinthus* L.	242,414,090	Shinay	Gum	Easy delivery	[42]
	*Pistacia atlantica* Desf. ex Batt.	250,077,142	Gonget	Gum & Oil	Aphrodisiac, vaginal infections, anemia, and back pain	[61]
Apocynaceae	*Nerium indicum* Mill.	200,018,423	Khar-zahreh	Leaf & flower	Abortion	[61]
	*Nerium oleander* L.	200,018,424	Kaner & Ganderay	Roots	Abortion	[58,63,70,75]
Asclepiadaceae	*Calotropis procera* (Aiton) W.T.Aiton	210,000,158	Tirkha pan/Pulhar pan, Ak, & Spalmay	Whole plant	Abortifacient promotes the expulsion of "dead blood" and lochia after delivery. Menstrual cramps, uterine complications, and Leucorrhoea are all symptoms of leucorrhoea	[13,42,63,78]
Berberidaceae Berberidaceae	*Berberis lyceum* Royle	242,420,754	Zerleg, Kwaray, & kashmal	Whole plant	Internal wounds, postpartum pains, waist pain, used as an astringent in gynecological disorders, Amenorrhea, pile, gonorrhea	[31,42,66,71]
	*Berberis orthobotrys* Bien. ex Aitch.	200,008,336	Ishkeen	Leaves, Fruit, Root, Bark	Broken limbs, fractures, skin healing following birth, blood pressure, and so on	[83]
Betulaceae	*Betula utilis* D.Don	200,006,168	Jongi	Bark	Backache, tonic for women after birth for a total of forty (40) days	[83]
Bignoniaceae	*Tecomella undulata* (Sm.) Seem.	250,071,457	Purpak	Stem and flowers	Abnormal menstrual cycle, and beneficial for sterile women	[64]
Bombacaceae	*Bombax ceiba* L.	242,420,835	Simbal	Roots, gum, & flower	Leucorrhea, amenorrhea	[66]
Boraginaceae	*Onosma hispida* Wall. ex G.Don	250,084,536	Arrelling	Whole plant	After delivery fever, internal cuts, and lochia, encourage the expulsion of "dead blood" and lochia.	[42]
Brassicaceae	*Brassica rapa* L.	N/A	Not Found	Leaf	Cure gynecological disorders and hepatitis A, B, and C.	[84]
	*Brassica campestris* L.	200,009,247	Sarson	Leaves	Mastitis	[70]
	*Capsella bursa-pastoris* (L.) Medik.	200,009,292	Kiseh keshish, & Bambesa	Whole plant	Irregular menstrual, sedative	[61,63,70]
	*Nasturtium officinale* W.T.Aiton	200,009,627	Termera, Tara mera, & Talmera	Leaves	During delivery relax uterus muscles, and sterility	[58,63,66,70,73]
	*Sisymbrium irio* L.	200,009,679	Khelikheli	Seeds	Pregnancy	[70]
Burseraceae	*Commiphora stocksiana* (Engl.) Engl.	250,063,166	Chandrhu	Leave & root	Backache Joint pain and bone fracture	[80]
Buxacaeae	*Sarcococca saligna* (D.Don) Muell.-Arg.	200,012,668	Shangal	Roots	Gonorrhea	[58]
Cannabaceae	*Cannabis sativa* L.	200,006,342	Banga	Leaves & bark	Gonorrhea and Pregnancy	[70]
Capparidaceae	*Capparis decidua* (Forssk.) Edgew.	250,063,266	Karir	Stem, Leaves, & fruit	Menstrual cramps are relieved, digestive worms are removed, it is used as a sexual stimulant, and it relieves flatulence.	[78]
Celastraceae	*Gymnosporia royleana* (Wall. ex Wight & Arn.) Benth. & Hook.f.	250,068,932	Pataki	Seed	Pregnancy	[70]
Chenopodiaceae	*Chenopodium ambrosioides* L.	N/A	Arunpale	Leaves	Delivery pain	[58]
	*Tanacetum parthenium* (L.) Sch.Bip.	242,101,204	Gul-e-daudi	Flowers	Help to cure abnormal menses	[58]
Colchicaceae	*Colchicum autumnale* L.	N/A	Zafran	Bulb	Leucorrhea	[66]
Compositae	*Carthamus tinctorius* L.	200,023,631	Poong & Kasum	Whole plant	Menses pain, back pain, and abnormal stoppage of menses	[31,58]
Convolvulaceae	*Convolvulus arvensis* L.	200,018,801	Perwatai & Pryvatay	Whole plant	Sexual debility, menses	[66,70]
	*Cuscuta reflexa* Roxb.	200,018,823	Asmanbotai, jamaldarai, & Maraz botay	Whole plant	strangulation, suffocation of the womb sterility	[42,58,63,75]
Cruciferae	*Lepidium sativum* L.	200,009,589	Tartizak	Seed	Aphrodisiac lactiferous, carminative, abortion, and menstruation additive	[61]
Cucurbitaceae	*Citrullus colocynthis* (L.) Schrad.	242,426,424	Gegelanjook, Maraginye, Truh, Tummaor, Korrtumma, Maraghone, Tuma, & Kakora	Whole plant	Pregnancy diabetes, abortifacients, mastitis, elevated blood sugar, painful menstruation, numbness or tingling in the legs, and digestive disorders, which facilitate the removal of "dead blood" and lochia after delivery	[42,58,61,63,70,78]
	*Momordica charantia* L.	200,022,698	Karella	Roots	Abortion	[13,58,63,70]
Cupressaceae	*Juniperus excelsa* M.Bieb.	N/A	Ubasht	Fruit	After arrival, encouraging the expulsion of "dead blood" and lochia	[42]
	*Thuja orientalis* L.	242,352,240	Cheelai	Leaves	Used in the excessive menstrual cycle	[85]
Cyperaceae	*Cyperus rotundus* L.	200,026,713	Delloca	Whole plant	Menses	[70]
Elaeagnaceae	*Elaeagnus angustifolia* L.	200,014,543	Gindawar, Shekarkuch, Shooto, & Hamamo	Whole plant	Stop bleeding during menses and hair care, Menses pain, and Cure back pain.	[31]
Ephedraceae	*Ephedra intermedia* Schrenk & C.A.Mey.	200,005,509	Oman	Stem	Internal injury, swelling of the uterus, fertility, and wounds	[42]
Equisetaceae	*Equisetum ramosissimum* Desf.	233,500,623	Jorter & Horsetail	Whole plant	Gonorhea	[70]
	*Equisetum ramosissimum* subsp. Debile	233,500,623	Satgandi booti	Whole plant	Gonorrhea	[58]
	*Equisetum arvense* L.	233,500,616	Bandakay	Whole Plant	Gonorrhoea	[63]
Ericaceae	*Rhododendron arboreum* Sm.	200,016,337	Rantol, Gul-e-nameer	Flower	Leucorrhoea	[58,63,70]
Euphorbiacae	*Mallotus philippensis* (Lam.) Müll.Arg.	242,413,649	Kamla & Kambela	Bark	Gonorrhoea	[63,66,70]
	*Euphorbia parviflora* F.Muell. ex Benth.	N/A	Ganda botay	Leaves	Leucorrhoea	[58,63,70]
	*Ricinus communis* L.	200,012,604	Arand, Jalabotukh, harnoli	Fruit, Seeds, & Roots	Stop the menses, leucorrhoea, constipation, Period pain, contraceptive, easy delivery, and abortion	[3,13,42,58,63,66,70,73,75]
Fagaceae	*Quercus dilatata* Lindl.	242,424,827	Toor banj	Fruit	Treat gonorrhea and urinary tract infections	[71]
	*Quercus infectoria* Olivier	N/A	Zarghoonm azoo	Fruit (Galls)	Waist pain and postpartum pain	[42]
Fumariacea	*Corydalis govaniana* Wall.	242,314,672	Mamera	Roots	Syphillis	[58]
Geraniaceae	*Geranium wallichianum* D.Don ex Sweet	242,323,331	Srazela, Ratanjot, & Sra zelay	Roots	Menses regulate, leucorrhoea, and tonic after delivery	[58,63,66,70,73,75]
Hypericaceae	*Hypericum perforatum* L.	200,014,237	Sheen chai	Fruit & Shoot	Menstrual irregularities, uterine prolapse	[66,70]
Juglandaceae	*Juglans regia* L.	200,006,112	Ghuz tree, Ghoz	Bark, fruit	Gynae and sexual tonic	[42,66,70]
	*Juglans regia* L.	200,006,112	Akhrot	Stem, leaves, Root, & Fruit coat	Strengthen the teeth after delivery	[42]
Juncaceae	*Juncus thomsonii* Buchenau	200,027,419	Gawag	Whole plant	Pregnancy	[66]
Lauraceae	*Cinnamomum camphora* (L.) J. Presl	200,008,697	Capur	Leaves	Abnormal stoppage of menses	[58]
Liliaceae	*Asparagus racemosus* Willd.	240,001,105	Satavari & Tendonay	Roots	Milk increase	[3,58,63,70,76,77]
	*Linum perenne* L.	250,063,157	Aalesi/Zaghar/azghar	Seeds	Internal cuts, injury, inflammation, and womb/uterus swelling	[42]
	*Linum usitatissimum* L.	200,012,411	Homans	Seed	Smooth delivery and cure back pain.	[31]
Loranthaceae	*Viscum album* L.	200,006,582	Neela	Berry	Sexual weakness	[58]
Lythraceae	*Woodfordia fruticosa* (L.) Kurz	200,014,668	Tahvi & Dhawai	Flowers	used for contraception and to help with the menstrual cycle in the surrounding area It's also used to avoid general body leakage and control menstrual cycles	[20,63,75,71,79]
Meliaceae	*Azadirachta indica* A. Juss.	220,001,427	Neem & Nim	Leaf & seeds	control irregular periods, stop excessive menstrual bleeding, emmenagogue	[58,71]
	*Melia azadirachta* L.	220,008,324	Bakana & Dhariak	Bark, Fruits gum, & Roots	Gonorrhea, leucorrhea, and emmenagogue	[49,58,63,70,75]
Menispermaceae	*Tinospora sinensis* (Lour.) Merr.	242,352,344	Praiwatay	Leaves & Stems	Sexual tonic	[66]
	*Tinospora sinensis* (Lour.) Merr.	242,352,344	Gilu	Roots	Irregular Menstruation	[63,[74], [75], [76]]
Moraceae	*Ficus carica* L.	200,006,351	Anzar	Fruit	Sexual weakness and leucorrhea	[66]
	*Ficus religiosa* L.	200,006,369	Peepal	Wood & Bark and fruit	Cure white discharge	[3,58,63,70,71,82]
	*Ficus benghalensis* L.	233,500,648	Bargad, Burr	Latex	Sexual weakness	[3,58,70,76]
	*Ficus glomerata* Roxb. ex DC.	242,322,248	Gular & Inzar	Fruit	Menorrhagia	[58,63,70]
Myrtaceae	*Eucalyptus globulus* Labill.	200,014,782	Lachi	Leaves, oil, & stem	Menses	[70]
	*Myrtus communis* L.	200,014,805	Mano	Leaves & Roots	Regulate the menses	[66]
	*Psidium guajava* L.	200,014,806	Amrud	Leaves & Bark	Expulsion of placenta	[58,63,70]
Neuradaceae	*Neurada procumbens* L.	220,009,223	Chhapri	Whole plant	Tonic for the reproductive organs and treatment for a general ailment	[78]
Nyctaginaceae	*Boerhavia coccinea* Mill.	242,414,731	Insut & Punara	Whole plant	Menses	[70]
	*Boerhavia procumbens* Mill.	250,070,600	Biskhipra & Mangotie	Whole plant	painful periods in women, Menstrual flow regulation	[78]
	*Boerhavia diffusa* L.	200,007,007	Biskhapra & Ensut	Roots	Bleeding after delivery	[3,20,58,63,66,70,[75], [76], [77]]
	*Mirabilis jalapa* L.	200,007,009	Guli abas	Roots & Leaves	Uterine waste, gonorrhea	[66]
Oleaceae	*Fraxinus xanthoxyloides* (Wall. ex G.Don) DC.	200,015,555	Toor	Bark, Stem, & Leaves	Gynae	[70]
	*Olea ferruginea* Royle	230,003,919	Khuna Tree, & Kao	Fruits & Leaves	Menses, Used for gonorrhea.	[70,79]
Oxalidacaea	*Oxalis corniculata* L.	200,012,375	Khatimithiboti & Trokay	Leaves	Vomiting	[58,63,70]
Paeoniaceae	*Paeonia emodi* Wall. ex Royle	200,008,033	Mamekh	Leaves	Regulation of menses, abdominal pain	[66]
Papaveraceae	*Papaver somniferum* L.	220,009,871	Posat & Kashkash	Flower, Fruit, & Seeds	Abortifacient, pregnancy, and tonic after delivery	[63,70]
Phyllanthaceae	*Phyllanthus emblica* L.	200,012,600	Alam & Lashora	Seed powder & Fruits	Aphrodisiac, leucorrhoea, vaginal cancer, and ovarian infection	[3,63,66]
Plantaginaceae	*Plantago ovata* Forssk.	250,095,355	Aspaghol	Seeds & Husk	Gonorrhea	[58,63]
	*Veronica agrestis* L.	N/A	Khoso beta	Whole plant	Regulate menses and pregnancy	[70]
Plumbaginaceae	*Plumbago zeylanica* L.	200,017,527	Chmchi pattar	Root	Abortifacient	[70]
Poaceae	*Desmostachya bipinnata* (L.) Stapf	200,025,171	Ghar chichona grass	Whole plant	Menses	[70]
	*Eleusine indica* (L.) Gaertn.	200,025,306	Not Found	Leaf	Prolapse uterus and treat menstruation if it takes a long time. Useful for curing gynecological problems, liver disorders, febrifuge, and blood dysentery.	[84]
	*Saccharum munja* Roxb. ex Fleming	242,426,431	Not Found	Not Found	Medicinal herbs used in birth control	[84]
	*Triticum aestivum* L.	200,026,445	Ghanam	Seeds	To keep the body warm after birth, strengthen the backbone, waist, and uterus	[42,63]
Polygonaceae	*Bistorta bistortoides* Pursh	250,060,031	Howar	Whole plant	Gonorrhea	[70]
	*Pteropyrum olivieri* Jaub. & Spach	242,100,140	Karwan kush	Leaves	Prevent excessive bleeding in menses	[64]
	*Rumex dentatus* L.	200,006,747	Khatembul	Leaves	Diuretic, astringent, and strengthen the stomach, also used for abortion	[81]
Portulacaeae	*Portulaca oleracea* L.	200,007,020	Marlai, Orkharay	Leaves	Slim the abdomen and gonorrhea	[42,63]
Primulaceae	*Androsace rotundifolia* Hardw.	210,000,059	Ratti booti	Leaves	Abnormal stoppage of menses	[58]
Punicaceae	*Punica granatum* L.	200,014,674	Beechil (tomaw), Anar	dried pericarp & Flowers	Menses pain, back pain, and leucorrhoea	[3,31,63,77]
Resedaceae	*Oligomeris linifolia* (Vahl) Tiegh.	200,009,755	Shootk & Shata	Seeds	Cure back pain, abnormal menstrual cycle, and motion of children, Slim the abdomen, swelling of the ovary, uterus	[42,64]
Rhamnaceae	*Ziziphus nummularia* (Burm.f.) Wight & Arn.	250,075,386	Jangli bair & Karkana	Roots	Abortion	[58,63]
Rosaceae	*Crataegus songarica* K.Koch	242,315,254	Ghonii tree	Leaves, Stem, & Bark	Gynae	[70]
	*Prunus amygdalus* Batsch	N/A	Badam	Seeds	Backache, strengthen the brain and weakness	[42]
	*Prunus domestica* L.	200,011,157	Alocha	Fruit	Menstrual irregularities and leucorrhea	[66]
	*Rosa moschata* Herrm. ex E.K.Ross	N/A	Gulab	Flower	Start menstruation and anti-constipation	[66]
	*Rubus niveus* Thunb. ex Murray	200,011,503	Pogana	Roots	Used in the excessive menstrual cycle	[85]
	*Spiraea tomentosa* L.	242,417,312	Not known	Flower	Easy delivery	[58]
Rubiaceae	*Jaubertia aucheri* (Boiss.) Dandy	220,006,942	Khartoosa	Seeds	Slim the abdomen	[42]
	*Randia tetrasperma* (Lam.) Merr.	242,424,856	Mainphal	Fruit	Abortion	[70]
	*Rubia cordifolia* L.	200,022,213	Chero	Roots	Flow of lochia	[58]
Rutaceae	*Citrus aurantium* L.	200,012,428	Nimbo	Fruit	Infection of the fetus	[66]
	*Zanthoxylum armatum* DC.	242,355,514	Timbar tree, & Laighunay timber	Fruit, leaves, Shoots & Roots	Abortifacient that is used to cure gonorrhea and diseases of the urinary tract	[70,71]
Salicaceae	*Salix acmophylla* Boiss. & Balansa	242,100,165	Chekar	Leaves, & Twigs	Menses	[70]
Sapindaceae	*Cardiospermum halicacabum* L.	200,013,187	Kanphuti	Roots	Amenorrhea	[58]
	*Dodonaea viscosa* (L.) Jacq.	200,013,193	Bansathra & Ghoraskay	Leaves	Excess menstrual flow	[58,63,70]
	*Sapindus mukorossi* Gaertn.	200,013,220	Raitha	Seeds	Easy delivery	[58]
Saxifragaceae	*Bergenia stracheyi* (Hook.f. & Thomson) Engl.	200,008,336	Bisabur & Sanspar	Leaves & roots latex	Headache, blood pressure, fatigue, vertigo, joint pain, backache, and immediate skin healing following birth are all signs of breastfeeding.	[70,83]
Simarubaceae	*Ailanthus excelsa* Roxb.	250,075,630	Darawa	Bark, leaves	As A tonic after delivery	[58]
Solanaceae*//	*Datura metel* L.	200,020,519	Barbaka	Whole plant	Gonorrhea	[70]
	*Datura stramonium* L.	200,020,519	Daturoo, & Daltora	Leaves	Inflammation of breast	[58,63,70]
	*Hyoscyamus niger* L.	200,020,521	Joli gao, Bhang lewana	Leaves & Seeds	Pregnancy and stopping too much bleeding after delivery	[42,70]
	*Solanum surattense* Burm.f.	230,005,939	Manraghonay/mahokri	Whole plant	Gonorrhea and Pregnancy	[70]
	*Solanum nigrum* L.	200,020,597	Mako & Kachmachu	Leaves	Menorrhagia	[58,63,70]
	*Solanum virginianum* L.	200,020,613	Mokri	Whole plant	Conception	[58]
	*Withania coagulans* (Stocks) Dunal	242,426,281	Panir & Panir doda	Fruits	Leucorrhoea	[70]
	*Withania somnifera* (L.) Dunal	200,020,616	Kotilal, Jangli paneer, & Asghand,	Whole plant	Leucorrhea, female impotency, menses, and sexual weakness	[3,13,49,58,63,70,73,75,82]
Tamaricaceae	*Tamarix aphylla* (L.) Karst.	200,014,298	Sheen ghazz	Leaves, & bark	Gynae	[70]
Tiliaceae	*Corchorus depressus* (L.) Peterm.	250,064,389	Bundairy/Mundairy	Whole plant	Cure gonorrhea and swelling of the urinary bladder	[64]
	*Grewia optiva* J.R.Drumm. ex Burret	242,422,703	Dhaman & Pastonay	Bark	Easy delivery	[58,63,70]
Urticaceae	*Urtica dioica* L.	220,014,002	Karry	Leaves	Regulate the menstrual period and stop bleeding	[81]
Valerianaceae	*Valeriana jatamansi* Jones ex Roxb.	200,022,557	Murma	Stem & roots	treatment of painful menstruation, hypertension, cramps, and irritable bowel syndrome	[71]
Verbenacea	*Phyla nodiflora* (L.) Greene	200,019,371	Makna	Whole plant	Cure skin diseases, after-birth diseases in women, swollen cervical glands and gastric problems, and puerperal fever after delivery	[58,84]
	*Verbena officinalis* L.	200,019,436	Koso beeta, karenta, & Shomakay	Whole plant	Pregnancy, menses, and miscarriage	[49, 58,63,70]
	*Vitex agnus-castus* L.	200,019,437	Gowanik	Whole plant	Useful for the menstrual cycle	[64]
	*Vitex negundo* L.	200,019,442	Not known	Roots	Regulate menstrual cycle	[63]
Zingiberaceae	*Curcuma longa* L.	200,028,370	Kurkoman, Halichi (Haldi Uudu)	Rhizome & stem	Internal injury following birth, external stitches, postpartum, infections, menses pain, and back pain	[31,42]
Zingiberaceae	*Zingiber officinale* Roscoe	200,028,468	Adrak	Roots	Blood pressure, regulate menses, wound healer, and pain killer after pregnancy	[13,63,66,73,77]
Zygophyllaceae	*Peganum harmala* L.	200,012,416	Spalani & Harmal	Whole plant	Easy delivery, Emotional problems, painful menstruation, seizures, insanity, and itchy eyes	[42,78]
	*Tribulus terrestris* L.	200,012,418	Bhukhra & Markundai	Leaves	Gonorrhoea	[13,58,63]

N/A = Not Available.

### 3.3 Plant parts used

**Fig. 3:**
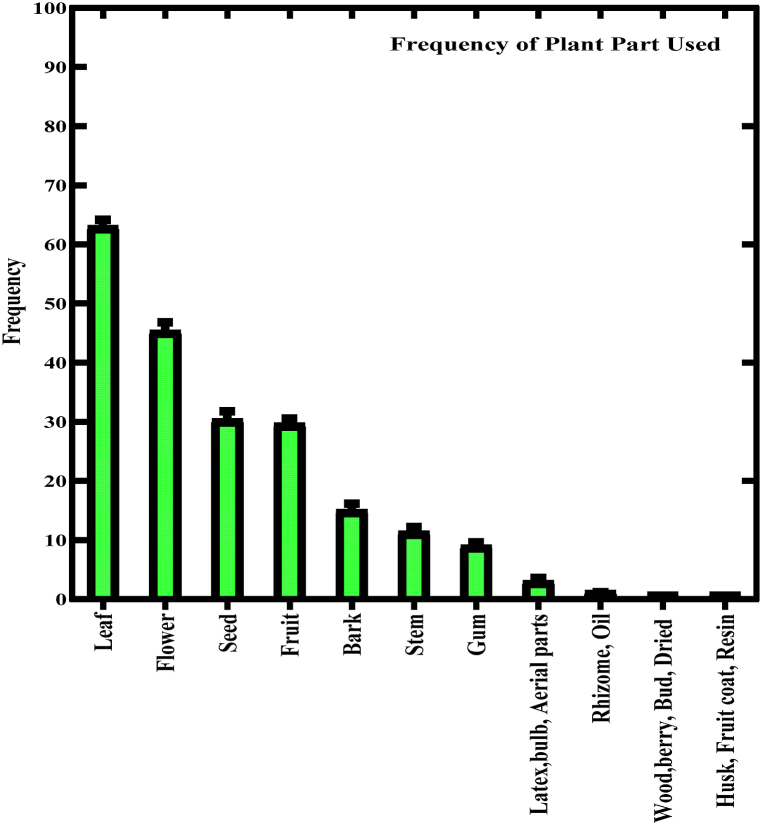
Frequency of study area plant parts used in traditional medicines.

Leaves, flowers, seeds, fruits, roots, bark, and stems were mostly used parts in rural communities of Pakistan with 29 %, 22 %, 14 %, 14 %, 13 %, 7 %, and 5 %, respectively as reported in the current review. Aerial parts, dried pericarp, bulb, bud, berry, latex, wood, rhizome, husk, fruit coat, oil, resins, twigs, and shoots were also used in minimum percentage (Fig. 3). In traditional medicine, various plant parts have been historically employed to address gynecological issues. Leaves from specific plants have found use in remedying menstrual disorders, and menopausal symptoms, and supporting overall reproductive health, while flowers of diverse plant species have been harnessed for their potential medicinal properties in managing menstrual discomfort and regulating menstrual cycles [86]. Additionally, certain seeds have been integrated into traditional practices to address female reproductive health concerns, including fertility issues and hormonal imbalances, whereas fruits have been valued for their nutritional contributions to women's well-being, particularly during pregnancy and lactation. Roots from particular plants have been incorporated into herbal formulations aimed at addressing uterine disorders and promoting women's health. Furthermore, the bark of specific trees has been utilized in traditional medicine for its potential medicinal properties, with potential relevance to gynecological issues. While less common, certain plant stems may also contain compounds with potential medicinal properties for gynecological health, though their usage may be limited [87]. It is important to emphasize that the efficacy and safety of these traditional remedies warrant further scientific investigation.

### 3.4 Role of bioactive compounds in gynecological problems

**Fig. 4:**
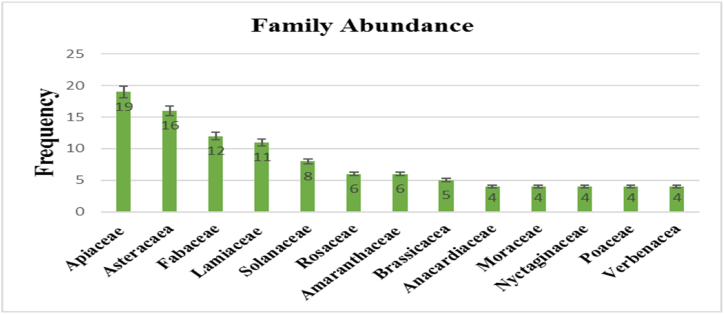
Dominant plant families used for the treatment of gynecological diseases.

Bioactive compounds, which are not essential for the body, possess the ability to influence one or multiple metabolic processes, leading to improved health. Consequently, functional diets have garnered increased attention as an alternative to traditional disease treatments. The medicinal and nutraceutical value of medicinal plants can be attributed to the presence of distinctive functional groups. Phytochemicals are naturally occurring chemical compounds in plants with diverse biological properties and therapeutic advantages [88]. For decades, natural bioactive compounds have served as traditional remedies for treating diseases. Recent scientific discoveries have unveiled the health benefits of functional biological active metabolites and shed light on their fundamental mechanisms of action. Phytochemicals play pivotal roles in disease prevention and treatment by thwarting oxidative damage and interacting with the immune system [89]. The current research highlights that different parts of certain plants contain a variety of secondary metabolites, which are valuable in combating gynecological diseases and can also be harnessed by local communities for the treatment of various other ailments. In the current study, Apiaceae, Asteracaea, Fabaceae, Lamiaceae, Solanaceae, Amaranthaceae, and Rosaceae are reported as dominant families, and their abundance is shown in Fig. 4.

**Fig. 5:**
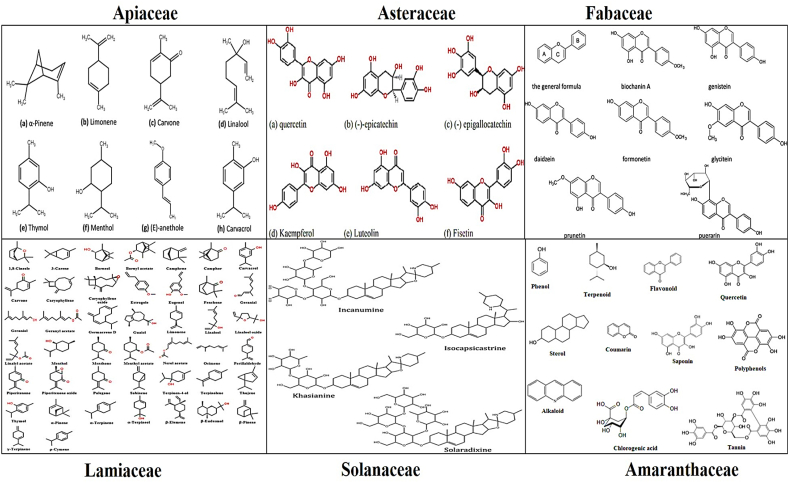
Chemical structures of phytochemicals responsible for treating gynecological complications.

Based on the literature review these are enriched with bioactive compounds [[90], [91], [92], [93], [94]]. Major bioactive compounds found in dominant families are presented in Table 1 and Fig. 5 shows the chemical structures of these compounds.

Medicinal herbs are rich sources of a diverse range of bioactive compounds, including steroids, flavonoids, polyphenols, tannins, saponins, glycosides, terpenoids, and anthraquinones. These compounds play a crucial role in providing therapeutic benefits for various gynecological disorders, such as polycystic ovarian syndrome, infertility, pubertal changes, postmenopausal syndrome, menopause, and low breast milk production [95]. The clinical effectiveness of these bioactive constituents lies in their ability to promote blood circulation and alleviate blood stasis, making them a widely utilized treatment for gynecological conditions characterized by blood stasis syndromes, such as QSBS primary dysmenorrhea [96]. Another study has highlighted the significance of bioactive compounds found in the Fabaceae family of plants for addressing polycystic ovary syndrome (PCOS), a prevalent metabolic disorder affecting approximately 1 in 10 women worldwide, particularly those of reproductive age [97]. PCOS is considered one of the most critical gynecological disorders in women. PCOS is characterized by elevated insulin levels due to insulin resistance, leading to increased levels of luteinizing hormone (LH) and follicle-stimulating hormone (FSH). In PCOS patients, the LH/FSH ratio is higher than normal. Plant-derived bioactive compounds have demonstrated their potential in managing diabetes and reducing cholesterol levels. By utilizing herbal plant extracts, it has been observed that the LH/FSH ratio, which typically stands at approximately 3.16 in PCOS patients, can be reduced to 1.61 [98]. Therefore, naturally occurring active substances found in herbs have been employed in medicinal practices for treating various ailments. Plant materials hold promise for the treatment of numerous diseases and have traditionally played a crucial role in global healthcare. The use of herbal remedies has gained popularity as they are perceived as safe with minimal or no side effects.

In the current study, we juxtaposed the traditional phytotherapeutic data with previously documented information on gynecological disorders in women as found in ethnobotanical literature, including studies by Refs. [75,[99], [100], [101]]. Our examination revealed that the plant species identified in this review were also utilized for addressing various other medical conditions. This underscores the necessity for thorough investigations into the medicinal properties of these presented plant species through pharmacological experiments. These traditional herbal remedies, when subjected to scientific authentication and clinical validation, have the potential to pave the way for the development of novel pharmaceutical drugs. The traditional phyto-therapeutical data gathered in the current study were contrasted with prior data on gynecological disorders in women present in ethnobotanical literature [[31], [32], [33], [34], [35], [36]]. It was revealed that the plant species presented in this review are also used in various diseases. There is a need to investigate the presented medicinal plants to find the rationality of their therapeutic uses through pharmacological experiments. These traditional herbal medicines led the way to potential drugs through scientific authentication and clinical verification. Medicinal plants play a crucial role as a primary source of herbal remedies for healthcare needs within rural communities [102]. Moreover, various studies found that trees, shrubs, and herbs were under pressure due to foolish use such as fodder, firewood, construction, agricultural instruments, etc, which led to the extinction of plant species [103,104]. Unchecked grazing and browsing were also contributing to this problem. The illegitimate looping for fuel and timber lumbering also causes a threat to the medicinal plant species [20,105]. There is a need to adopt appropriate measures for the conservation of biodiversity to control future trouble. In Table 2, we mentioned a vast variety of traditionally used medicinal plants and their parts for the treatment of women's diseases, and Table 3 is about graphical representation of plants that help in accurate identification.

**Table 3:**
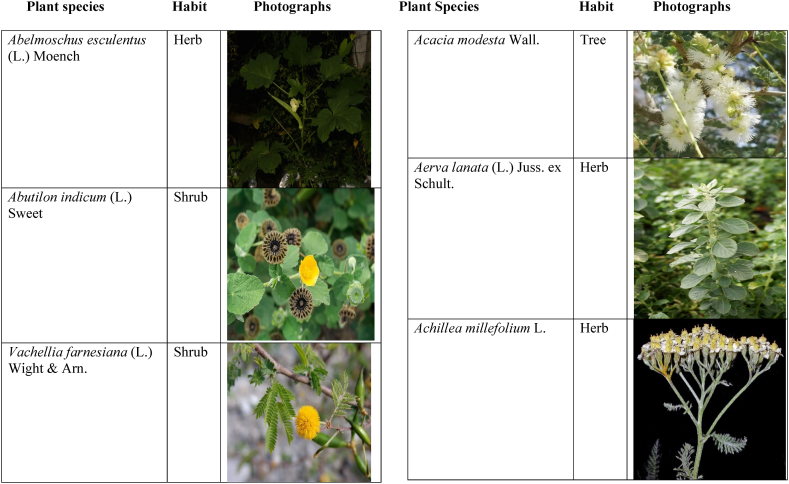

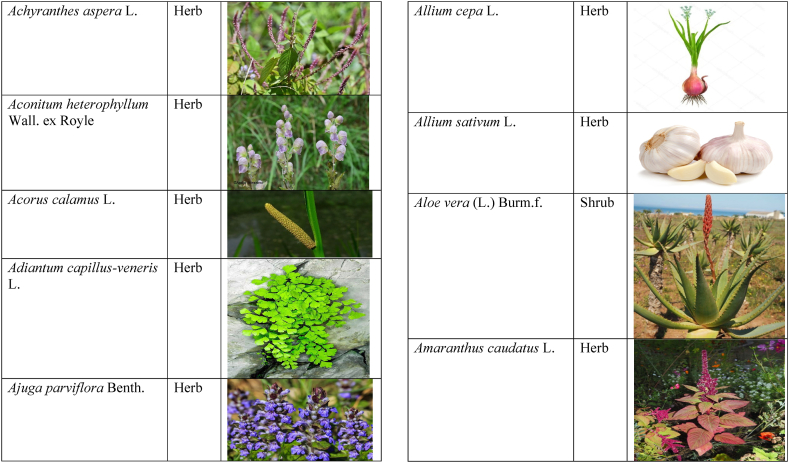

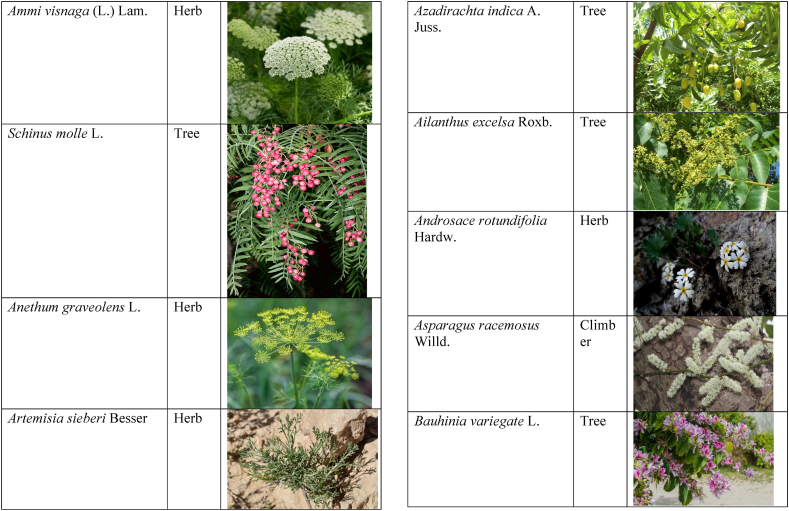

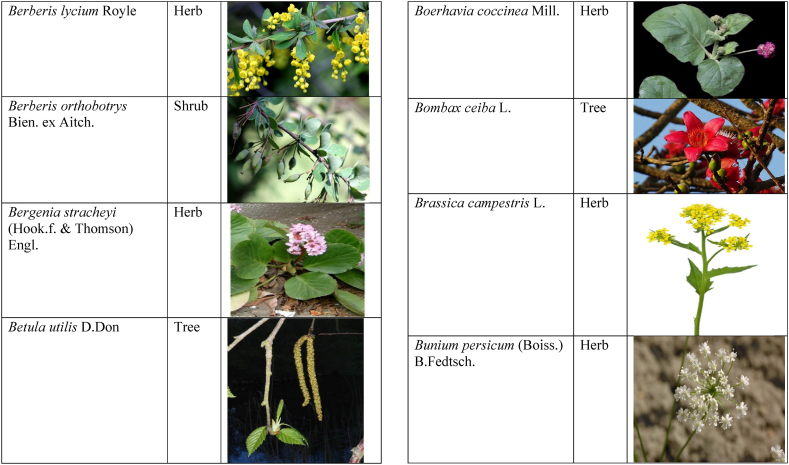

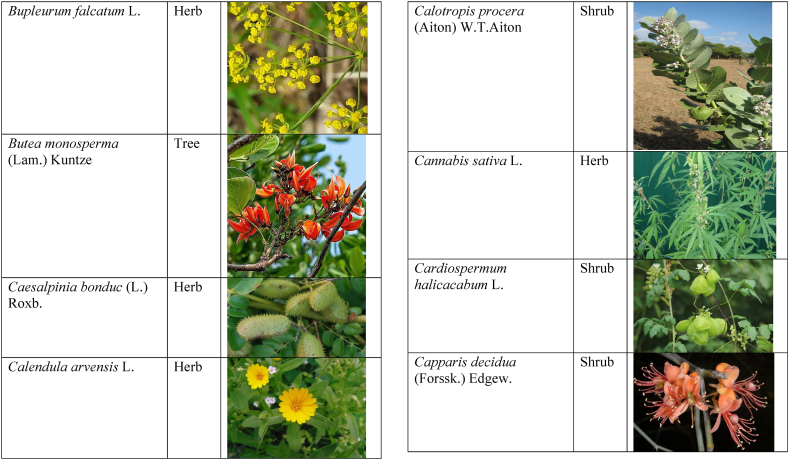

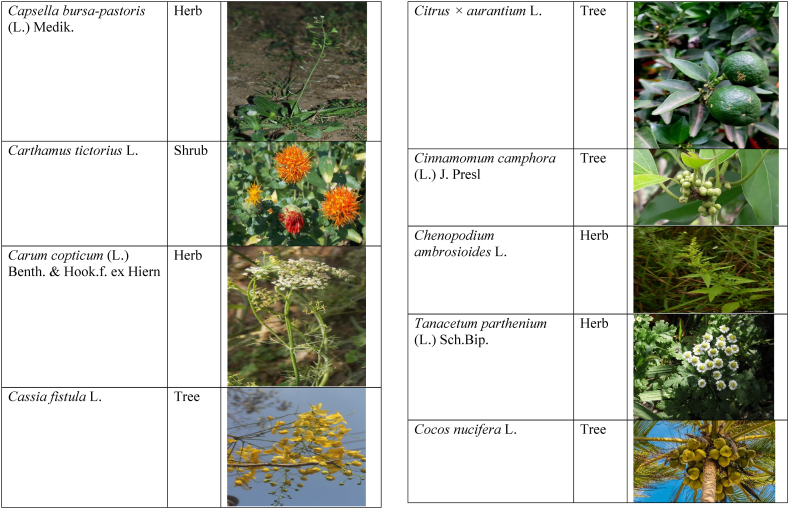

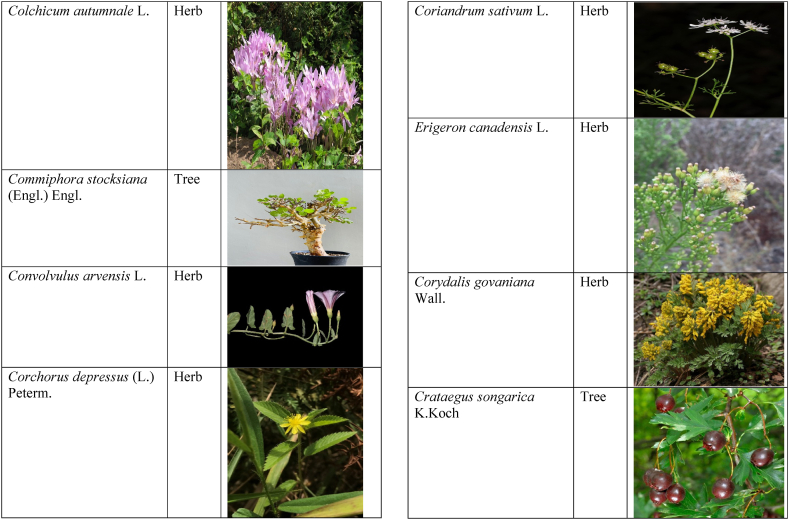

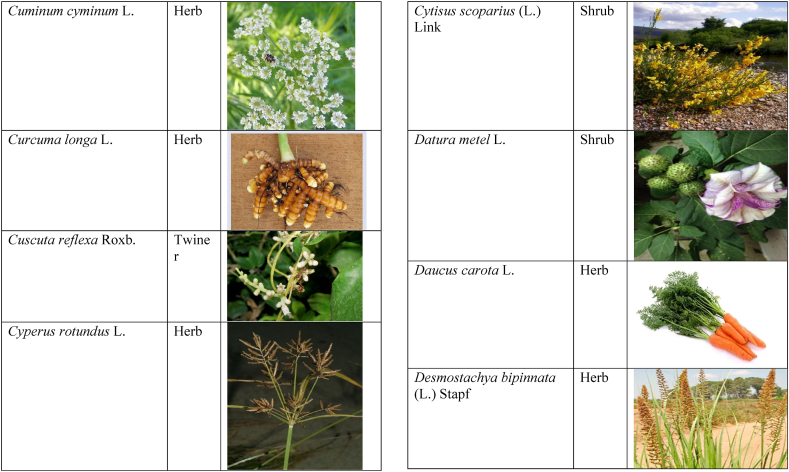

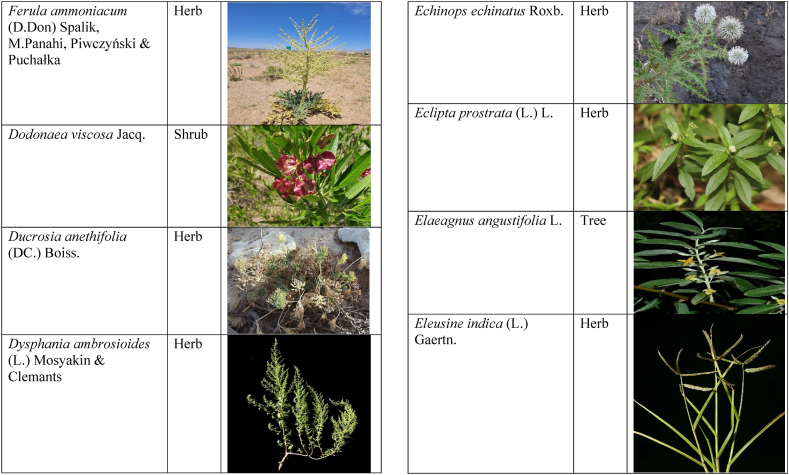

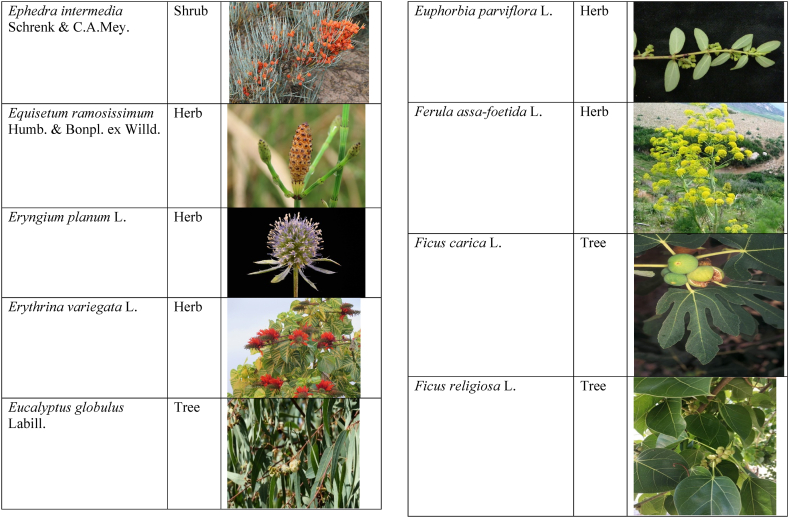

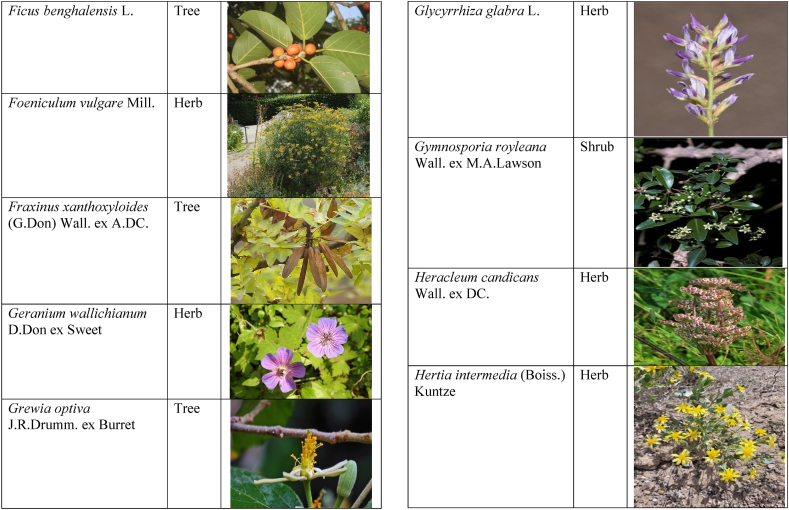

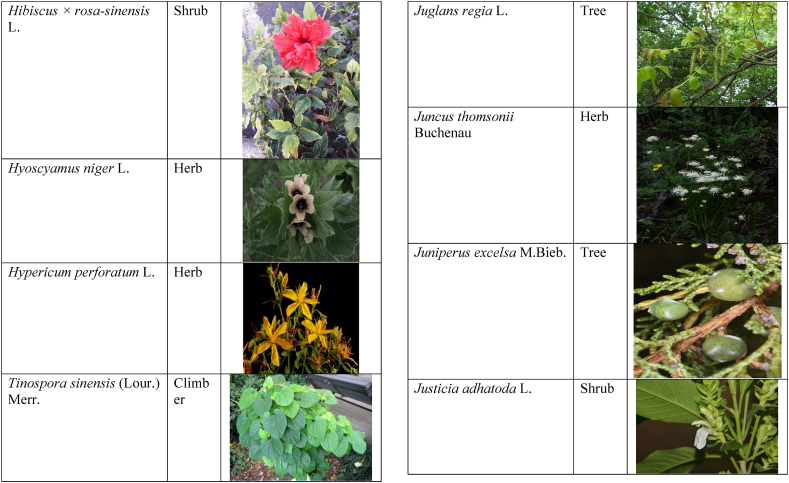

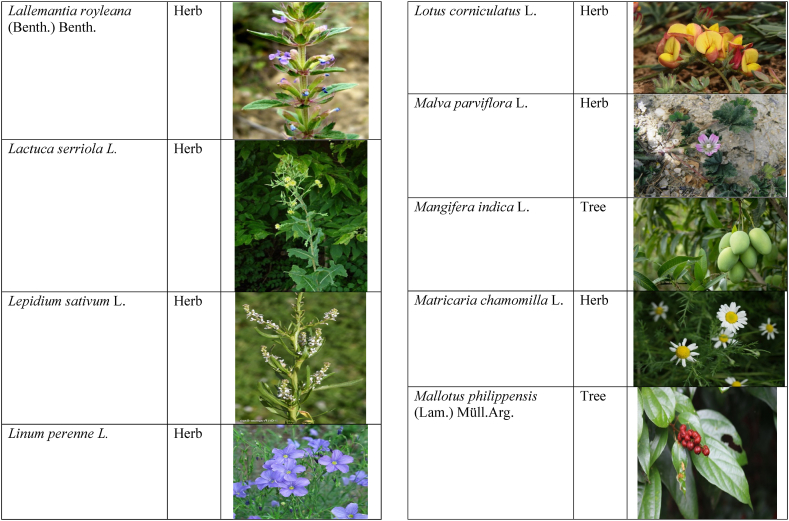

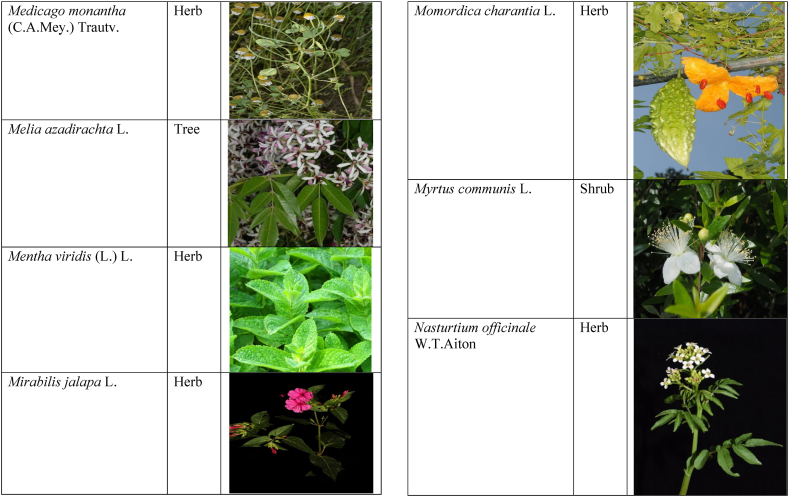

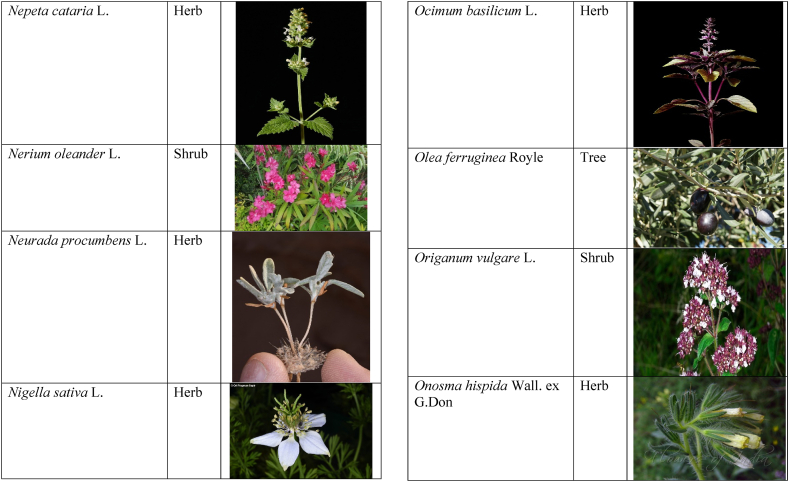

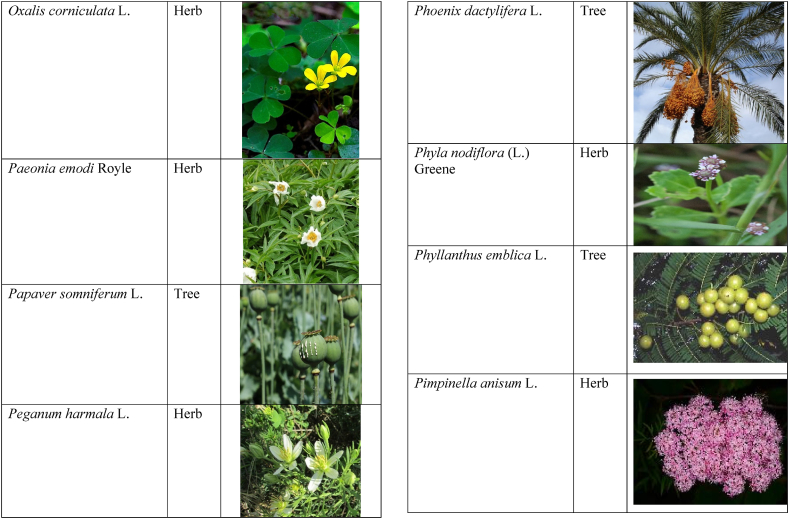

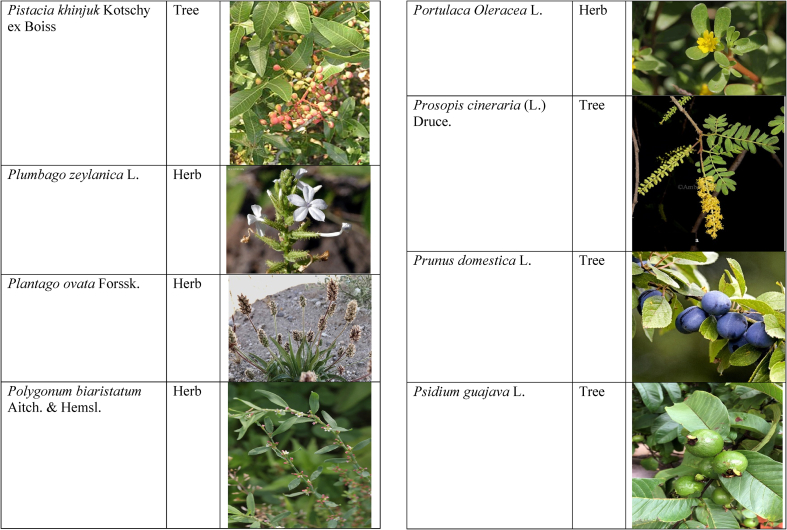

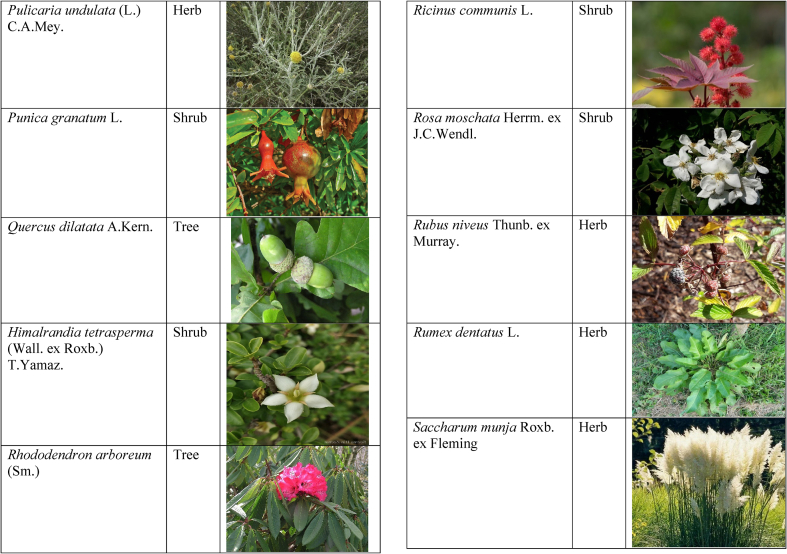

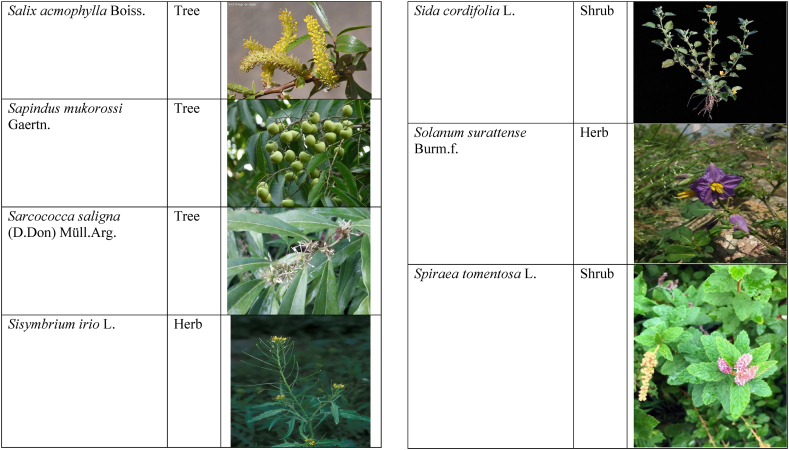

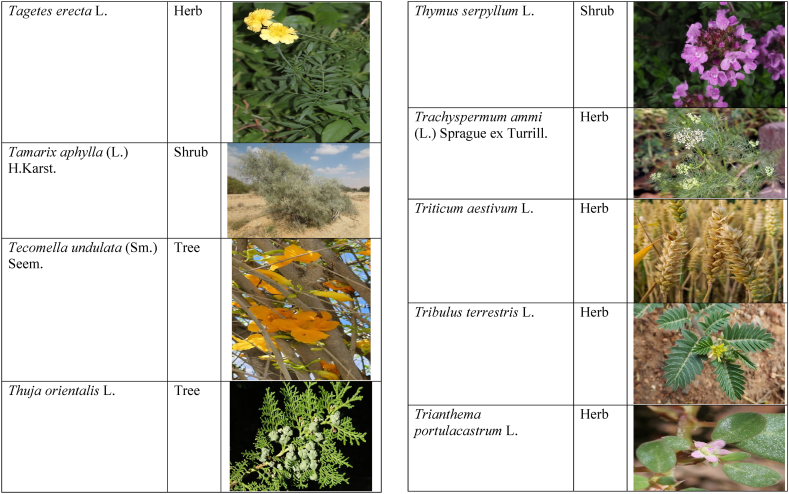

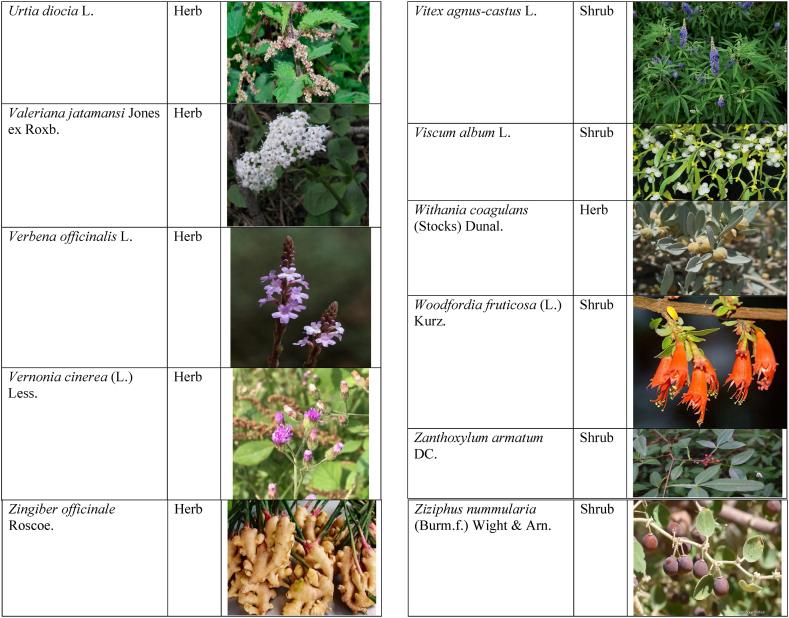
Photographical representation of traditionally used plant species and their habit for gynecological disorders.

## 4 Conclusion

The focus of this study was on how medicinal plants are used to cure women's diseases in rural Pakistani societies. This study paper described 217 plant species belonging to 89 families that are widely used to treat several women's ailments such as fatigue, change of moods due to menses problems, gonorrhea, pregnancy-related problems, food cravings, inflation throbbing pain in the breast, cramps in the abdominal and pelvic region, excessive white discharge, mastitis, irritability, abortion and headache, uterine hemorrhage, Menorrhagia, Amenorrhea, Menopause, Vomiting Abortion, infertility/lactation, the flow of lochia and other issues linked to pregnancy. Still, there is a need of lot work on the biological, and pharmacological activities and toxicity of these species. Safety controls and specific screening of natural results are also required. Older women have a stronger connection to native plants and may be more knowledgeable about their therapeutic properties. Younger generations have a closer association with allopathic medicines, which causes the knowledge of traditional remedies to decline. Furthermore, the future preservation of the medicinal flora will benefit from this study. The data present in this review provide a gateway for researchers to utilize it and form new molecules. Also, study the isolated chemicals obtained from these plants used in the development of natural drugs and provide benefits to the health.

## Ethical approval

Not required.

## CRediT authorship contribution statement

**Salihah Khadim:** Writing – original draft, Validation, Software, Resources, Project administration, Methodology, Formal analysis, Data curation. **Khafsa Malik:** Writing – review & editing, Supervision, Project administration, Investigation, Formal analysis, Data curation, Conceptualization. **Abeer Kazmi:** Writing – review & editing, Writing – original draft, Software, Formal analysis, Data curation, Conceptualization. **Tahira Sultana:** Writing – review & editing, Writing – original draft, Visualization, Validation, Supervision, Methodology, Investigation, Formal analysis, Data curation, Conceptualization. **Amir Ali:** Writing – review & editing, Writing – original draft, Visualization, Validation, Software, Methodology, Investigation, Formal analysis. **Khalid Mehmood:** Writing – review & editing, Validation, Supervision, Resources, Project administration, Formal analysis, Data curation, Conceptualization. **Rizwan Ul Hassan:** Writing – review & editing, Writing – original draft, Validation, Methodology, Data curation. **Muhammad Nasir Bashir:** Writing – review & editing, Writing – original draft, Visualization, Validation, Supervision, Software, Resources. **M. Mahmood Ali:** Writing – review & editing, Supervision, Resources, Project administration, Methodology, Investigation, Funding acquisition, Formal analysis, Data curation, Conceptualization.

## Declaration of competing interest

The authors declare that they have no known competing financial interests or personal relationships that could have appeared to influence the work reported in this paper.
